# *Akkermansia muciniphila* Delays the Progression of Diabetic Nephropathy by Reducing the Accumulation of Uremic Toxins in the Feces and Peripheral Blood

**DOI:** 10.2174/0118715303343599250210103212

**Published:** 2025-03-20

**Authors:** Xixi Song, Mingyan Yao, Jing Zhang, Bo Huang, Xin Li, Mengjuan Zhang, Jingqiu Cui

**Affiliations:** 1 Department of Endocrinology and Metabolism, Tianjin Medical University General Hospital, Tianjin, China;; 2 Department of Endocrinology and Metabolism, Baoding No.1 Central Hospital, Baoding, China;; 3 Department of Cardiology, Affiliated Hospital of Hebei University, Hebei, Shijazhuang, China

**Keywords:** Gut microbiota, diabetic kidney disease, *Akkermansia muciniphila*, uremic toxins, diabetic nephropathy, DKD model

## Abstract

**Background:**

The pathogenesis of diabetes and diabetic kidney disease (DKD) is closely related to the imbalance of gut microbiota. We aimed to explore whether exogenous *Akkermansia muciniphila* (*A. muciniphila*) supplementation affected the progression of DKD.

**Methods:**

The feces from normal subjects, diabetic patients without kidney diseases, and patients with DKD were collected, and the changes in microbial communities were analyzed. A rodent db/db DKD model was also constructed to investigate whether the abundance of *A. muciniphila* would be altered in response to renal function decline. The measurement of inflammatory cytokines in peripheral blood, fecal short-chain fatty acids (SCFAs), renal histopathology, and Western blot analysis were carried out to further evaluate the effects of *A. muciniphila*.

**Results:**

The relative abundance of *A. muciniphila* was found to be lower in the feces of DKD patients and also in the intestine of DKD mice. After exogenous supplementation of *A. muciniphila*, the levels of urinary protein, blood urea nitrogen, and creatinine decreased in DKD mice, as well as uremic toxins, trimethylamine-N-oxide (TMAO), p-cresol sulfonate, and indole sulfonate. *A. muciniphila* supplementation also increased the SCFAs gut production. The supplementation also protected the integrity of the intestinal mucosa by increasing MUC2, occludin, and zona occludens 1 (ZO-1) protein expression in the intestinal wall.

**Conclusion:**

Exogenous supplementation of *A. muciniphila* improved the imbalance of gut microbiota, reduced systemic inflammation, decreased uremic toxins, and protected the integrity of the intestinal mucosa, thus delaying DKD progression.

## 1 INTRODUCTION

Diabetes is a common systemic metabolic disorder that may, ultimately, cause diabetes kidney disease (DKD). The clinical manifestations of DKD include persistent albuminuria and/or the decline of glomerular filtration rate (GFR). As the disease progresses, DKD can be manifested with dominant proteinuria, edema, hypertension, and renal function damage, and further development may lead to end-stage renal failure [1]. Although the pathogenesis of DKD is still unclear, most studies report that changes in renal hemodynamics caused by hyperglycemia and a series of consequences caused by abnormal glucose metabolism are the basis of kidney dysfunction. Long-term hyperglycemia caused by insulin metabolism in patients with diabetes is the most critical cause of DKD, and the activation of many growth factors and cytokines directly influences the formation of lesions in the kidney [2, 3].

It has been found that metabolic diseases, immune diseases, gastrointestinal diseases, and even psychiatric diseases are closely related to the human microbial community. Metagenome-wide association study (MWAS) analyses reported significant differences in the structure and function of gut microbiota between healthy people and type 2 diabetic patients (T2D). The gut microbiota of healthy people is rich in microbiota diversity and can promote the production of beneficial metabolites, such as short-chain fatty acids (SCFAs) or vitamins. SCFAs are absorbed by intestinal epithelial cells and bind to receptors to induce Treg differentiation, thereby inhibiting inflammatory reactions and promoting tissue damage repair, which is beneficial for maintaining intestinal integrity and energy balance [4, 5]. Moreover, SCFAs also promote the secretion of glucagon-like peptide 1 (GLP1) and peptide YY, thus regulating blood glucose balance and controlling food intake [6]. The abundance of these bacterial communities and functions in T2D patients is significantly lower than in normal individuals. Thus, the change in gut microbiota in T2D patients is related to metabolic disorders and inflammation.

In the kidney, toxic derived metabolites produced by intestinal microorganisms cause damage and loss of renal podocyte, affecting the function of the glomerular basement membrane, which ultimately leads to increased urinary protein content [7]. The destruction of the intestinal epithelial barrier and hypoxia caused by high glucose trigger local and systemic inflammation and leukocyte inflow, which further accelerates the transfer of urinary toxins in the systemic circulation of DKD patients and animals [8, 9]. *A. muciniphila* is one of the strains of gut microbiota that colonize the mucus layer and colon by degrading and utilizing mucin as energy. The abundance of *A. muciniphila* strains increases to 1-4% of the intestinal microflora with the growth of human beings, and the SCFAs produced by the bacteria through mucoprotein degradation play an important role in human health [10]. It has been previously demonstrated that supplementation with *A. muciniphila* ameliorates chronic kidney disease-induced interstitial fibrosis in rats [11]. Therefore, the current study aimed to explore whether exogenous regulation of the abundance of gut microbiota, especially *A. muciniphila*, could delay the progress of DKD.

## 2 METHODS

### 2.1 Subjects and Fecal Samples

To clarify the effect of DKD on inducing microbial dysbiosis, feces were collected from three groups of subjects for 16s rDNA sequencing of gut microbiota. Each group contained 12 subjects, including 12 healthy controls, 12 diabetic patients without kidney disease, and 12 newly diagnosed DKD patients who had not taken treatment medication. To avoid the influence of food styles, we only enrolled local people in the study, and immigrant citizens from other regions were not included. Meanwhile, to avoid differences caused by sampling, the collected fresh feces were mixed with sterile rods and stored at -20°C for sequencing. All protocols followed the method previously reported by Di Segni, *et al.* [12]. The DNA of the microbiota was extracted using MoBio PowerSoil^®^ DNA Isolation Kit (Cat number: 200059-790, MO BIO Laboratories, Carlsbad, USA). The TruSeq Nano DNA LT Library Prep Kit (Cat number: 20015964, Illuminas, San Diego, USA) was used for library preparation and sequencing analysis, following the reference provided by the manufacturer [13]. The TruSeq^®^ was vertically resected, and the contents SBS Kit v3-HS (Cat number: FC-401-3002 Illumina) was used for paired-end sequencing of 2×300 bp on the MiSeq machine (Illumina). Based on the abundance spreading of operational taxonomic units (OTUs) in dissimilar samples, the variety of each sample was evaluated. Consequently, the structure of each sample at distinctive sorting was explored. The expression of *A. muciniphila* was measured by routine quantitative polymerase chain reaction (qPCR), as previously reported by Collado *et al.* [14]. The adopted primers were forward 5′-CAGCACGTGAAGGTGGGGAC-3′; reverse 5′- CCTTGCGGTTGGCTTCAGAT-3′ for *A. muciniphila*, and forward 5′-TCCTACGGGAGGCAGCAGT-3′ and reverse 5′-GGACTACCAGGGATCT AATCCTGTT-3′ for the bacterial 16s rRNA gene. The relative abundance of *A. muciniphila* was calculated by comparing the ratio of *A. muciniphila* 16S copies to total bacterial 16S copies.

The study was approved by the Medical Ethics Committee of Baoding First Central Hospital, and the approval number was (2022)-004.

### 2.2 DKD Mice Model and Administration of *A. muciniphila*

To clarify the effect of microbial metabolites on DKD and their impact on renal damage, a db/db background DKD mice model was constructed. The 8-week-aged db/db mouse was fed with high-sugar and high-fat diet (45% carbohydrate, 15% protein, and 40% fat, Beijing Orange Co., Beijing, China) for 2 months to aggravate hyperglycemia and kidney damage, and DKD was confirmed by positive urinary protein in the test paper. The age-matched C57BL/6J was used as a negative control with 8 mice in each group. The *A. muciniphila* (ATCC BAA-835) was purchased from the Guangdong Institute of Microbiology. The strain was cultured in brain heart infusion broth (Cat number: 024053; HuanKai Microbial, Guangzhou, China) supplemented with mucoprotein (1g/L) using an anaerobic incubator containing 5% CO_2_, 85% N_2_, and 10% H_2_. The cultured strain was mixed with aseptic saline into 1 × 10^8^ CFU suspension, and 0.3 mL suspension was administered daily by gavage after urinary protein was confirmed. The mice in the DKD model group received the same dose of physiological saline by gavage. The blood and urine were collected every two weeks to examine several biochemical indicators, including blood glucose, creatinine, urea, nitrogen, triglycerides, and total cholesterol in serum by commercial kits purchased from the Nanjing Jiancheng Bioengineering Institute (Nanjing, China). 24-hour proteinuria was tested by ELISA (Abcam, USA) [15], and the body weight of mice was estimated weekly. All mice were sacrificed by decapitation 2 months after gastric gavage, and the left kidney tissues were collected for histopathological observation. For colony forming units (CFU) measurement in the intestinal mucosa, 0.3 to 0.5 cm of the cecum was vertically resected, and the contents in the cecum were removed and gently rinsed with 0.01 mol/L phosphate-buffered saline (PBS). The homogenate of samples was diluted serially by physiological saline and finally added to the culture medium for cultivation and counting the CFU of each sample.

The animal study was approved by the Animal Ethics Committee of Tianjin Medical University (approval number: TMUaMEC2022047). All protocols followed the guidelines of the Institutional Animal Care and Use Committee.

### 2.3 Measurement of Inflammatory Cytokines in Peripheral Blood

The blood sample was collected from the posterior orbital vascular plexus after mice were anesthetized by 5% chloral hydrate (300 mg/kg, injected intraperitoneally). After the collected samples were left overnight at 4°C, the samples were centrifuged at 1000 g for 20 minutes at 4°C. The supernatants were collected, and serum IL-6, IL-1β, and TNF-α were measured using ELISA commercial kits (Cat number: E-EL-M0044c, E-EL-M0037c, and E-EL-M3063; Elabscience, Wuhan, China). According to the provided instructions, the diluted standard solutions or test samples with different concentrations were loaded in 96-well plates. Afterward, the diluted biotinylation antibodies, color-developing solution, and termination solution were added. The light absorption value of 450 nm (A450) was detected in the microplate reader (FACSCalibur flow cytometer, BD Biosciences, NJ, USA). Each sample was assayed in triplicate. The results were expressed in ng/ml.

### 2.4 Renal Histopathological Analysis

Renal tissues were fixed with 4% paraformaldehyde (PFA) for 24 h at room temperature. The tissues were cut into 6-μm paraffin sections following routine protocols. The sections were stained successively with 0.5% periodate, periodic acid-Schiff (PAS), and hematoxylin at 35˚C for 10, 15, and 1 min, respectively. The renal histopathological changes were observed under a light microscope with 10 high-power (magnification: x400) fields. The observed changes in tubules included cellular necrosis, loss of brush border, cast formation, vacuolization, and tubule dilation. The observed glomerulus injury included the changes in capillary and mesangium, and the alterations in proximal convoluted tubules were also observed. Lesions were classified into four severity categories: no lesions, segmental glomerulosclerosis, diffuse sclerosis, and exudative lesions. The proportion (%) of each category was calculated. The vacuolar degeneration of proximal tubular epithelium was characterized semi-quantitatively as one of the following four categories: absent, mild, moderate, and severe [16]. A total of 10 randomly selected field samples per animal were analyzed, and all eight animals from each group were included.

### 2.5 Gut microbe-derived Metabolites Measurement

TMAO is a gut microbe-derived metabolite and is a potential risk factor for many chronic diseases [17]. The plasma TMAO and its precursor, trimethylamine (TMA), were measured by liquid chromatography coupled to tandem mass spectrometry (LC/MS/MS). Detailed measurement protocol followed the method reported by Wang *et al.* [18]. The Acquire UPLC BEH HILIC (2.1 X 100 mm, 1.7 µm; Cat number: 186003461, Waters, Milford, USA) chromatographic column was used and connected to the ACQUITY UPLC BEH C18 VanGuard Pre-column (2.1× 5 mm, 1.7 μm; Cat number: 186003975, Waters). The flow rate was 0.5 mL/min, and the injected volume was 1.5 μL. The mobile phase elution gradient was generated by solvent A (ammonium formate in water, 10 mmol/L, with 0.1% formic acid) and solvent B (ammonium formate in 95% acetonitrile, 10 mmol/L, with 0.1% formic acid). The analytes were separated from 3% A linearly to 60% A over 4.5 min and then back to 3% A.

The p-cresol sulfate, indole, and indoxyl sulfate in plasma and feces were measured by the liquid chromatography- mass spectrometry instrument consisting of the 1260 infinity II binary pumps (Agilent, Santa Clara, USA) and 6460 triple quadrupole MS system (Agilent). Five microliters of serum samples were injected into a Kinetex Phenyl-Hexyl chromatographic column (Cat number: 00B-4500-AN; Phenomenex, Torrance, USA) with a flow rate of 0.4 mL/min. The gradient elution was 10 mmol ammonium formate in water (solvent A) and methanol (solvent B). The elution ratio of solvent B starts from 0% linearly to 40% over 2.7 min, then linearly to 85% B at 2.5 min, holds for 3 min, and then back to 40% B at 8 min. The Jet Stream ESI ion source was used in liquid chromatography-mass spectrometry with a sheath gas flow rate of 11 L/min. The temperature of the sheath gas was set at 350˚C; the speed and temperature of drying gas were 5 L/min and 300˚C. The auxiliary gas pressure was 45 psi, and the capillary voltage was 3.5 kV to -3 kV. The concentration of TMAO, p-cresol sulfate, indoxyl sulfate in plasma, and indole, TMA, and p-cresol sulfate in feces were all expressed as μM.

### 2.6 Fecal Short-chain Fatty Acids (SCFAs) Detection

The measured SCFAs [19] included acetic acid, propionic acid, and butyric acid. The mice were placed in a disinfected and clean cage before fecal collection. Then, 0.1 g of fresh feces was mixed with 1 mL of sterile physiological saline in a sterilized EP tube. After fully shaken, the solution underwent ultrasound in the ice water bath for 30 minutes, kept at 4˚C for 30 minutes, and then centrifuged at 13000 g at 4˚C for 15 minutes. The supernatant of fecal bacteria was taken, and 500 μl ethyl acetate was added for extraction. The solution underwent ultrasound in the ice water bath for 10 minutes and centrifuged at 13000 g at 4˚C for 10 minutes. The SCFAs in supernatant were analyzed by liquid chromatography-electrospray ionization-mass spectrometry (LC-ESI-MS/MS). Chromatographic conditions were UPLC BEH HILIC (Waters), chromatographic column; solvent A (0.1% formic acid in water) and solvent B (0.1% formic acid in acetonitrile); injected volume 2 μl. Mass spectrometry conditions were QTRAP™ 6500+ LC-MS/MS system (SCIEX, Framingham, USA); negative ion mode; curtain gas (CUR), 35 psi; collision gas (CAD), medium; ion spray voltage (IS) -4.5 kV; temperature (TEM) 450˚C; ion source gas1 (GS1): 40; ion source gas2 (GS2): 40. The results were expressed as μM in plasma and μg/g in kidney tissues.

### 2.7 Western Blot Analysis

The RIPA lysate (Cat number: R0010; Solarbio Life Science, Beijing, China) containing protease inhibitors was added to small intestine samples. The tissue was ground on ice and centrifuged to extract protein. The protein concentration was measured by the BCA method. A total of 30 μg protein was separated by polyacrylamide gel electrophoresis. The proteins were transferred to a PVDF membrane. The membrane was blocked by 5% skimmed milk for 12 hours, and then cultured with primary antibody of IL-6 (Cat number: 12912; Cell signaling technology), TNF-α (Cat number: 11948; Cell signaling technology), IL-1β (Cat number: 12242; Cell signaling technology), Muc2 (Cat number: PA5-103083; 1:1000; Invitrogen, Carlsbad, USA), Occludin (Cat number: ab222691; 1:1000; Abcam, Waltham, USA), ZO-1 (Cat number: ab276131; 1:1000; Abcam), and β-actin (Cat number: ab170325; 1:5000; Abcam) at 4°C overnight. The membrane was washed three times with tris buffer and then incubated at room temperature for 1 h with a secondary antibody (Cat number: ab205719; 1:5000; Abcam). The proteins were developed by ECL luminescent liquid (Cat number: SW2010; Solarbio life science) and exposed by Doc XR+ System (Bio-Rad, Hercules, USA). The grayscale of proteins was analyzed. The results were expressed as the relative expression of control (C57BL/6J mice).

### 2.8 Statistical Analysis

The data were presented and statistically analyzed by GraphPad 9.0 software. The measurement data were expressed in mean ± standard deviation (mean ± SD). The comparison between the two groups of normally distributed data was analyzed by independent sample t-test, and ANOVA was used for multiple groups comparison. Mann-Whitney U test was carried out between the two groups, and the Kruskal-Wallis (K-W) test was conducted for multiple groups comparison if data are non-normal distributed. *P*<0.05 was considered as statistically significant.

## 3 RESULTS

**Fig. (1):**
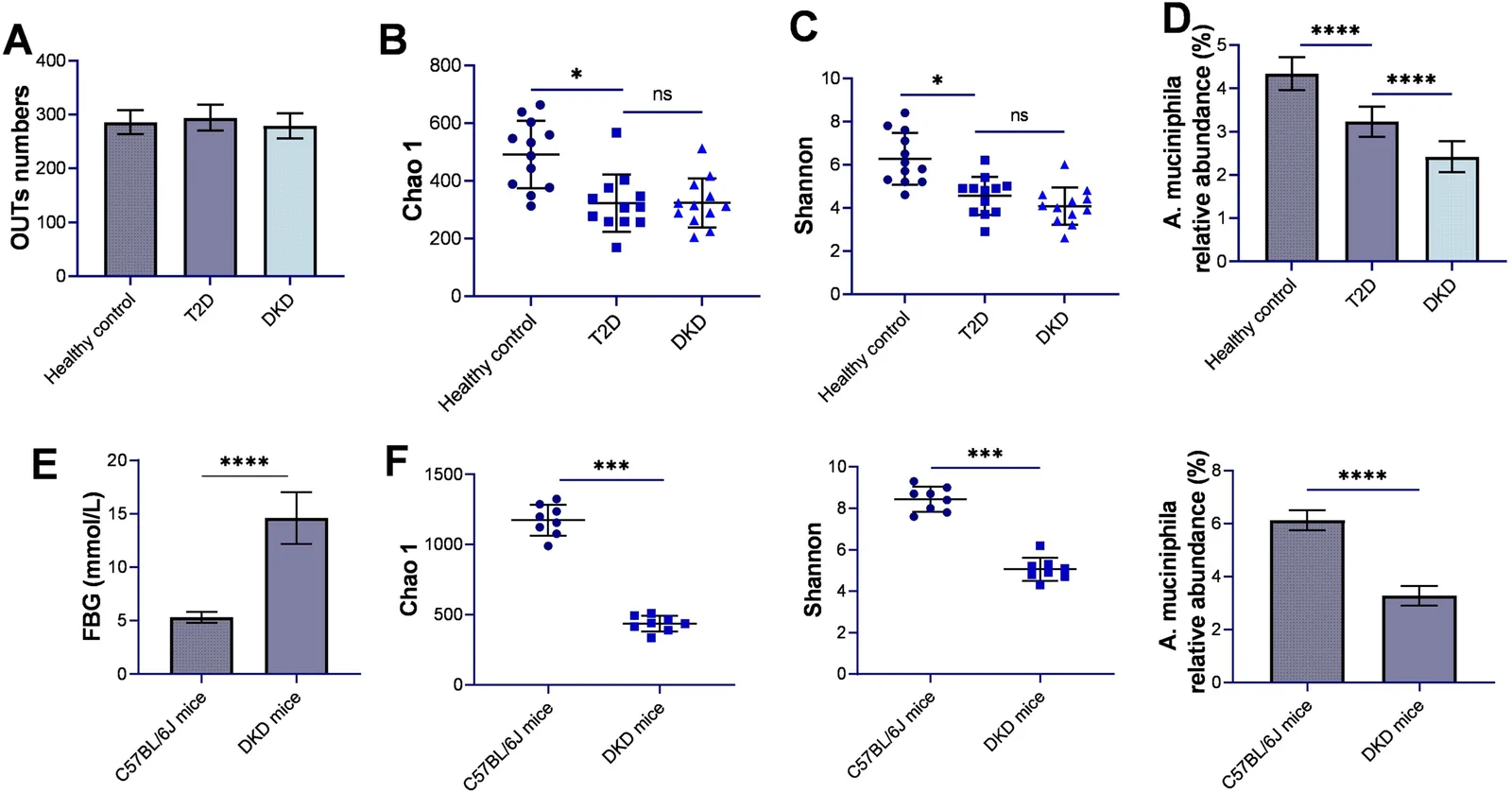
*A. muciniphila* decreases in DKD patients and DKD mice. (**A**). The OTUs number in the healthy control, T2D, and DKD groups (N=12 in each group); (**B, C**). The α diversity richness index (Chao1) and the diversity index (Shannon) decreases in T2D and DKD patients; (**D**). the *A. muciniphila* abundance in the intestines of patients with T2D and DKD reduced significantly; (**E**). DKD mice had significantly higher fasting blood glucose compared with healthy wild-type mice (C57BL/6J). (**F**). the diversity index and *A. muciniphila* abundance in DKD mice significantly decreased compared to healthy wild-type mice (C57BL/6J) (N=8 in each group). The K-W test was adopted in DKD patient’s analysis, and the Mann-Whitney U test was used between C57BL/6J and DKD mice. *, *p*<0.05; ***, *P*<0.001; ****, *P*<0.0001.

The demographic data of the included subjects are listed in Table **1**. The operational taxonomic units (OTUs) analysis results are shown in Fig. (**1A**). The OTUs numbers in the healthy control, T2D, and DKD groups had no obvious difference. The α diversity richness index (observed specifications, Chao1, Fig. **1B**) and the diversity index (Shannon, Fig. **1C**) showed a downward trend in the abundance and diversity of intestinal microbiota in patients with T2D and DKD. The qPCR was carried out to detect *A. muciniphila* in collected samples. Compared with healthy control subjects, the *A. muciniphila* abundance in the intestines of patients with T2D and DKD was significantly reduced in DKD patients (Fig. **1D**). DKD mice had significantly higher fasting blood glucose compared to healthy wild-type mice (C57BL/6J) (Fig. **1E**). Further analysis of the abundance of intestinal microbiota (α diversity, Chao1) and the diversity index revealed that *A. muciniphila* abundance in DKD mice significantly decreased compared to C57BL/6J mice (Fig. **1F**).

**Fig. (2):**
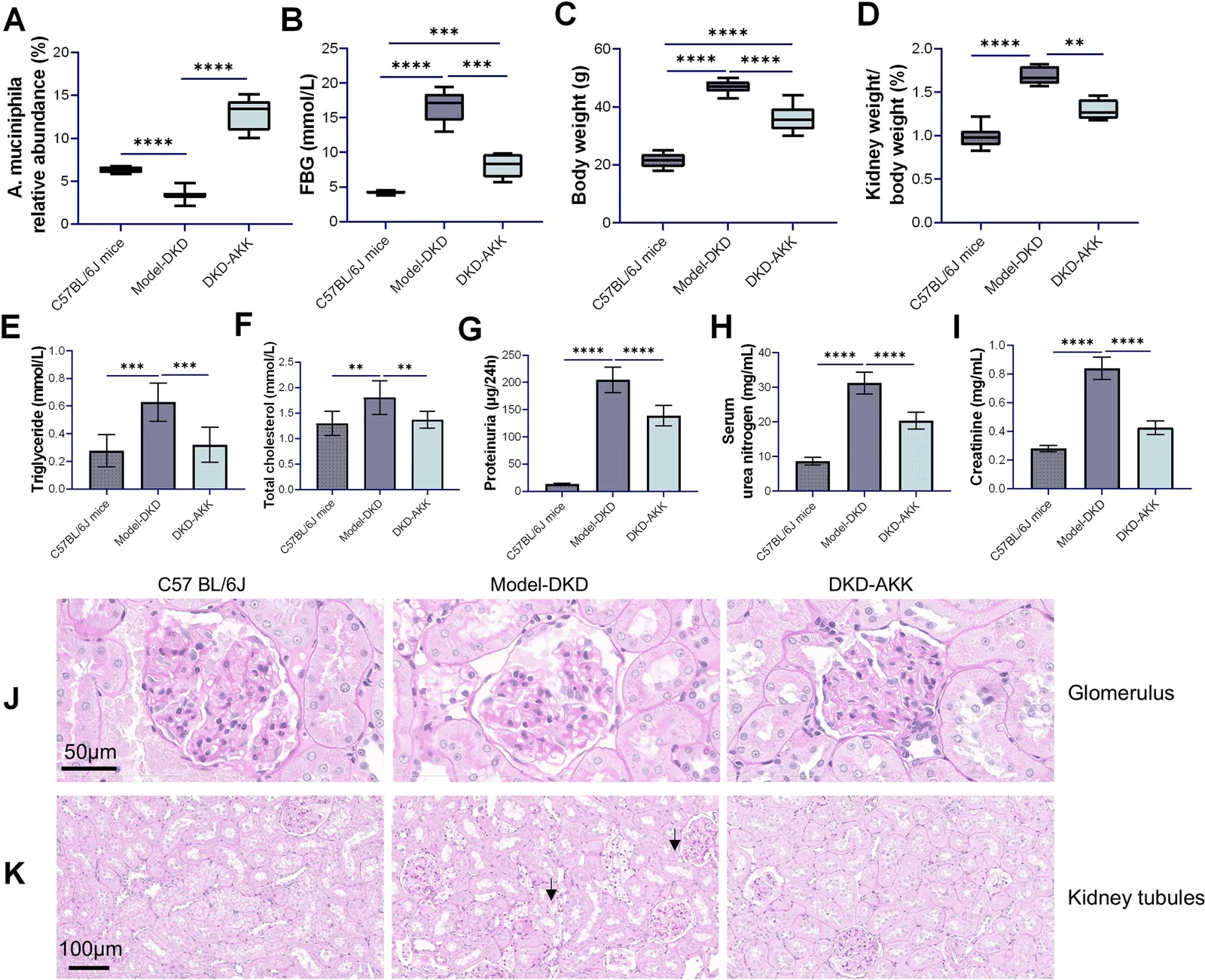
*A. muciniphila* improved body weight and blood glucose levels in DKD mice. **A**: The abundance of *A. muciniphila* in DKD mice increased after oral administration. **B**: *A. muciniphila* improved FBG level in DKD mice. **C**: *A. muciniphila* decreased the body weight of DKD mice. **D**: *A. muciniphila* decreased the proportion of kidney weight in DKD mice. **E-I**: *A. muciniphila* reduced triglyceride, total cholesterol, urine protein, serum urea nitrogen, and serum creatinine in DKD mice. **J-K**: *A. muciniphila* attenuated the destruction of glomerular and tubular structures caused by high glucose toxicity. Arrows refer to tubular injuries. N=8 in each group. Data was analyzed by ANOVA. **, *p*<0.01; ***, *P*<0.001; ****, *P*<0.0001.

Oral administration of *A. muciniphila* significantly increased its relative abundance in DKD mice, which was confirmed by qRT-PCR (Fig. **2A**). The fasting blood glucose (FBG) in the model group was significantly higher than in C57BL/6J mice. *A. muciniphila* implantation demonstrated a hypoglycemic effect in DKD mice (Fig. **2B**) presumably related to promoting glucose metabolism. *A. muciniphila* could decrease the body weight (Fig. **2C**) and reduced relative kidney weight (calculated as the ratio of kidney weight to body weight of mice) in DKD mice (Fig. **2D**). Moreover, *A. muciniphila* significantly reduced plasma triglycerides, total cholesterol, urine protein, serum urea nitrogen, and serum creatinine in DKD mice (Figs. **2E**-**I**). The pathological results also showed that *A. muciniphila* attenuated glomerulosclerosis (Fig. **2J**, Table **2**) and tubular injury (Fig. **2K** Table **3**) due to high glucose toxicity.

**Fig. (3):**
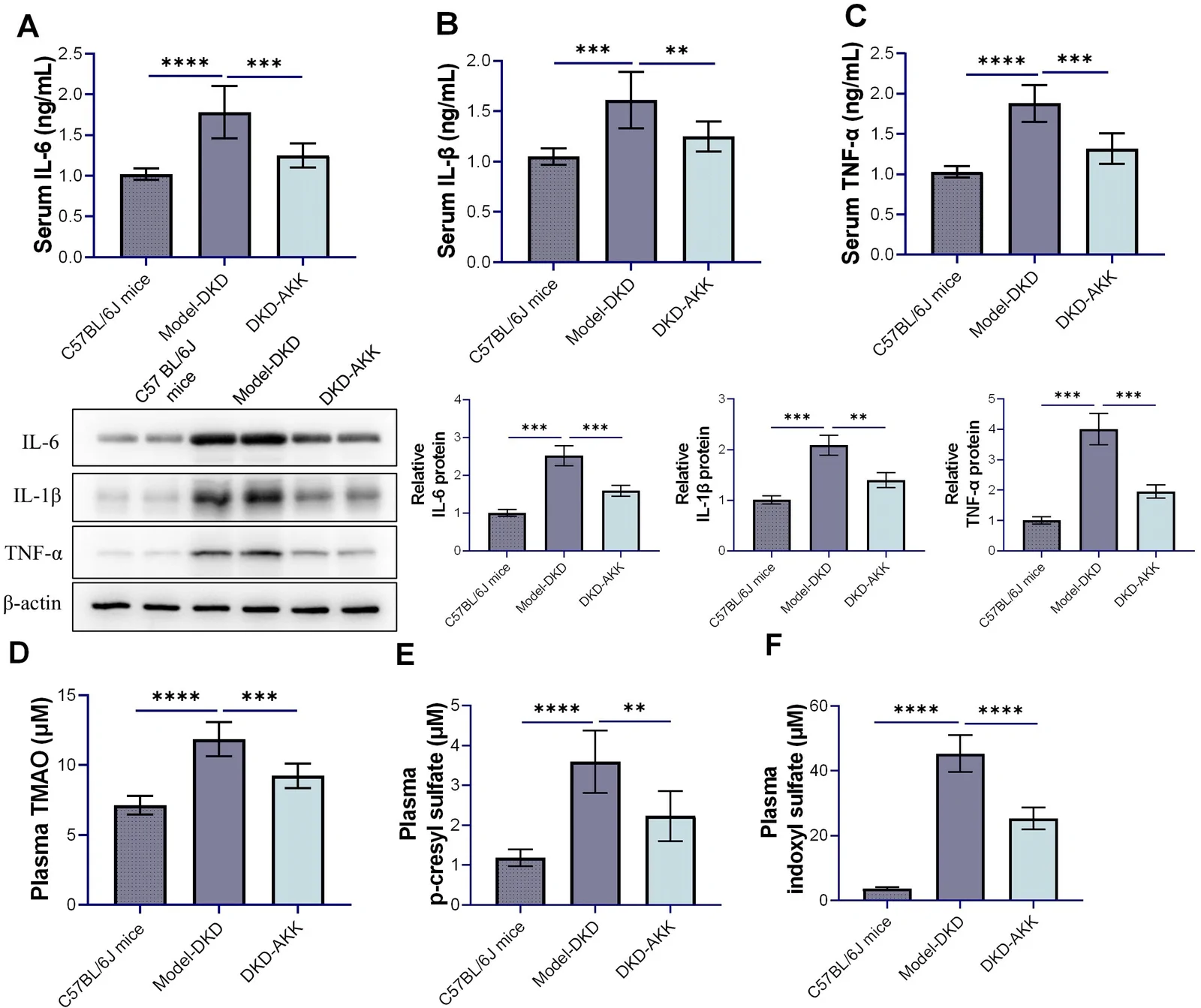
*A. muciniphila* attenuated the systemic and renal local inflammation of DKD mice. (**A-C**): Inflammatory factors IL-6, IL-1β, and TNF-α reduced after *A. muciniphila* supplementation. (**D**): Western blot analysis confirmed decreasing protein levels of IL-6, TNF-α, and IL-1β in the kidney after supplementation (n=3 in each group). (**E-G**): *A. muciniphila* attenuated the accumulation of uremic toxins TMAO, p-cresol sulfonate, and indole sulfonate in plasma. Data was analyzed by ANOVA. **, *p*<0.01; ***, *P*<0.001; ****, *P*<0.0001.

High glucose causes an increase in systemic inflammatory status. *A. muciniphila* administration significantly reduced IL-6, IL-1β, and TNF-α plasma levels (Figs. **3A**-**C**). These results are consistent with western blot analysis (Fig. **3D**), showing the parallel decrease in IL-6, TNF-α, and IL-1β protein expression in kidney tissue. Moreover, the accumulation of uremic toxins, TMAO, p-cresol sulfonate, and indole sulfonate, in plasma reduced after *A. muciniphila* supplementation (Figs. **3E**-**G**). All these results suggested that *A. muciniphila* supplementation attenuated the systemic and local renal inflammatory responses in DKD mice.

**Fig. (4):**
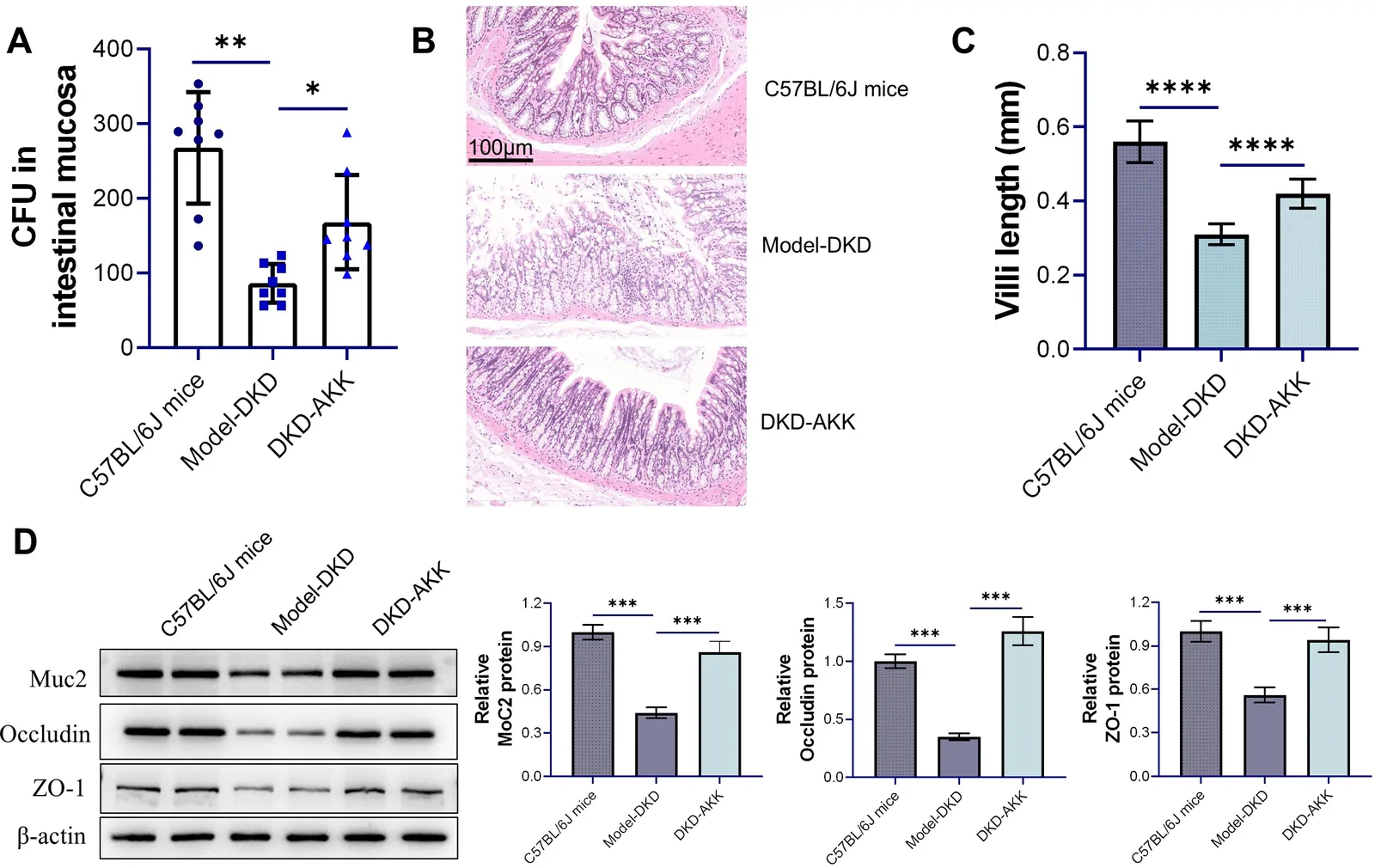
*A. muciniphila* improved injured intestinal mucosa in DKD mice (n=8). (**A**). muciniphila increased CFUs numbers of intestinal mucosa in DKD mice. (**B, C**): *A. muciniphila* colonization protected the integrity of the intestinal mucosa in DKD mice, promoted the repair of damaged mucosa, and increased intestinal villi length. (**D**): *A. muciniphila* increased the expression of tight junction protein MUC2, occludin, and ZO-1 in the intestinal wall of DKD mice (N=3). Data was analyzed by ANOVA. *, *p*<0.05; **, *p*<0.01; ***, *P*<0.001; ****, *P*<0.0001.

The gut dysbiosis could impair the barrier of the intestinal mucosa, cause pathogenic bacteria to translocate to the circulation, and further induce chronic inflammation. The results demonstrated that *A. muciniphila* supplementation increased CFU numbers in the intestinal mucosa (Fig. **4A**). Further pathological analysis showed that *A. muciniphila* supplementation protected the integrity of the morphological structure of the intestinal mucosa in mice, promoted the repair of damaged mucosa, and increased intestinal villi length (Figs. **4B**-**C**), thereby probably reducing systemic inflammatory reactions. Consistently, the reduction in MUC2, occludin, and ZO-1 protein expression in the intestinal wall of DKD mice was reversed by *A. muciniphila* supplementation (Fig. **4D**).

**Fig. (5):**
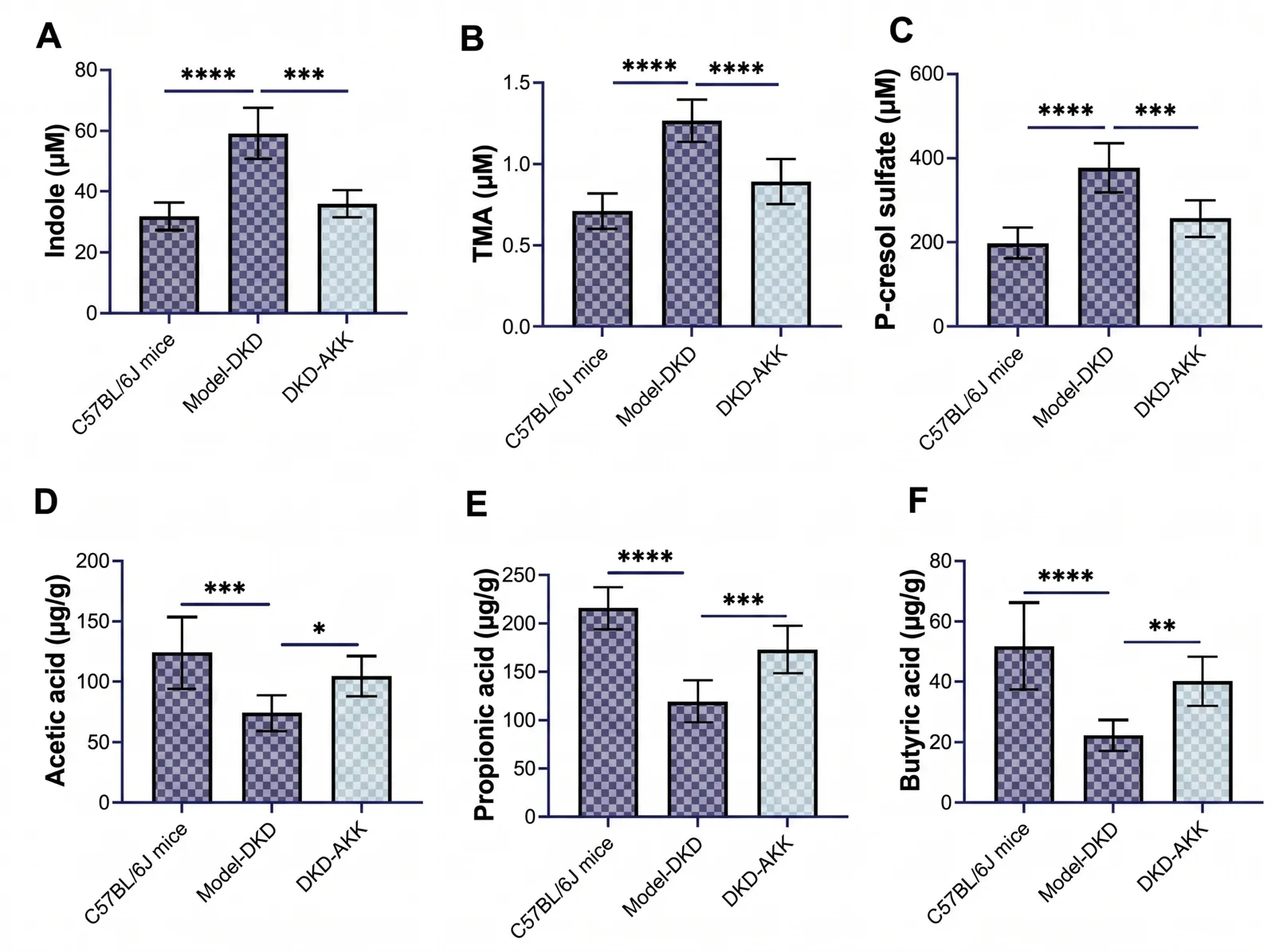
*A. muciniphila* regulated the production of intestinal toxins and SCFAs in the gut (n=8). (**A-C**): *A. muciniphila* decreased fecal indole, TMA, and p-cresol sulfonate in DKD mice. (**D-F**): *A. muciniphila* increased the level of fecal SCFAs acetic acid, propionic acid, and butyric acid. Data was analyzed by ANOVA. *, *p*<0.05; **, *p*<0.01; ***, *P*<0.001; ****, *P*<0.0001.

The fecal indole concentration was measured in mice from each experimental group. As shown in Fig. (**5A**), *A. muciniphila* supplementation significantly reduced indole production in feces. Meanwhile, *A. muciniphila* also decreased TMA and p-cresol sulfate in feces (Figs. **5B** and **C**). On the other hand, SCFAs, including acetic acid, propionic acid, and butyric acid (Figs. **5D**-**F**), were all significantly increased by *A. muciniphila* supplementation.

## 4 DISCUSSION

In this study, we found that exogenous *A. muciniphila* supplementation can attenuate the high glucose-induced kidney damage, alleviate the systemic inflammatory state of DKD, reduce the levels of adverse metabolites produced by gut microbiota, and, finally, delay the progress of DKD. There is still a considerable gap between the current treatment methods for DKD and clinical needs. Given the important role of intestinal microbiota, regulating gut microbiota may become a novel strategy for the treatment of DKD.

T2D is related to inflammation and gut microbiota disorder. Improving gut microbiota can improve T2D and reduce the risk of complications [20]. DKD is an important complication affecting the life and health of diabetic patients. Poorly controlled blood sugar might cause about 30%-40% of T2D patients to progress to DKD and even to end-stage renal disease (ESRD), which will bring a huge economic burden to society and seriously endanger the patient’s life and health [21]. Various clinical and animal studies have demonstrated the crucial role of gut microbiota in health maintenance and disease pathogenesis [22]. By altering the biochemical and biophysical environment of the intestine, late-stage chronic kidney disease can lead to disruption of the intestinal epithelial barrier and microbial imbalance. Recent studies have elucidated the impact of chronic kidney disease on the gut microbiota, pointing towards the kidney-gut axis. Chronic kidney disease has been proven to lead to the biological imbalance of intestinal microbiota [23, 24]. The harmful bacterial metabolites can be detected in DKD patients, while beneficial metabolites are reduced [25].

Compared with healthy people, the change in gut microbiota in patients leads to the proliferation of harmful metabolic bacteria and the reduction in beneficial metabolic bacteria, which is specifically manifested in the reduction of SCFAs-producing bacteria. Moreover, it promotes the expansion of harmful substances, such as indoxyl sulfate and p-cresol sulfate, endotoxin, and TMAO [26, 27]. Previous reports revealed that serum levels of indoxyl sulfate and p-cresyl sulfate increase parallel to GFR declination [28]. Colonic microorganisms are responsible for protein fermentation. Ammonia and urea are products of protein catabolism, where α- NH_3_ nitrogen (amino acids and intermediates) can be converted into phenols and indoles. Indole is generated by the bacterial metabolism of tryptophan in the colon, which is further oxidized and sulfurized into indoxyl sulfate in the liver. P-cresol sulfate is the product of phenol metabolism, and TMAO is the final product of choline and carnitine metabolism. Under normal circumstances, these substances in the blood circulation are excreted by the kidneys, but in the case of renal insufficiency, they accumulate and accelerate the progression of kidney disease [23].

The intestinal tract has a mechanical barrier, mucus barrier, immune barrier [8], and biological barrier, which maintain the stability of the intestinal tract and even the whole internal environment. Among them, the biological barrier is mainly composed of a large number of bacteria, viruses, protozoa, and fungi that parasitize on the surface of the intestinal cavity [29]. Additionally, the intestine is an important site for glucose absorption and impacts glucose homeostasis [30, 31]. Absorption of glucose in the small intestine physiologically contributes to the regulation of blood glucose levels. Therefore, promoting the integrity of the intestinal mucosa helps to reduce hyperglycemia caused by T2D or DKD. Moreover, intestinal microbiota and bile acid metabolism are directly related, and regulating the gut microbiome is also a direct factor in promoting blood glucose reduction [32]. Under normal circumstances, the intestinal microecology is in dynamic equilibrium with the body. Once this balance is disturbed, it may cause damage to the entire intestinal barrier function, leading to changes in the number and proportion of bacteria or spatial transfer, thus causing endogenous infection [29]. Chronic inflammation is common in patients with T2D and chronic kidney disease. Moreover, chronic inflammation is also the key factor determining the development of DKD. Bacteria and bacterial products, such as lipopolysaccharides, translocate from the intestinal cavity into the blood, causing typical low-grade inflammation in T2D and chronic kidney disease [8]. Accordingly, in a recent report [33], Sanchez-Alamo indicated that elevated IL-6 levels are associated with the progression of DKD in patients with T2D. IL-1β, which is an innate immune pro-inflammatory cytokine, also contributes to kidney fibrosis [34]. Finally, stimulation of TNF-α promoted the expression of renal inflammatory proteins [35] and regulated gut permeability [8]. In addition, the damage to an intestinal barrier in DKD patients leads to a decrease in tight junction protein expression and an increase in intestinal permeability. Upregulation of occludin alleviates the increased gut permeability induced by high cytokine levels [33]. In this regard, Feng *et al.* [34] demonstrated that the tight junction proteins, including ZO-1, occludin and claudin-1, decreased in nephrectomized DKD rats, while attenuating chronic kidney disease restored these levels.

The exact mechanism of the T2D-induced DKD has not been well elucidated, and it is currently believed that oxidative stress and inflammatory response play an important role in its pathogenesis and progression [35]. DKD patients exhibit endotoxemia, intestinal mucosal barrier damage, and translocation of intestinal bacteria and lipopolysaccharides into the blood, which may lead to chronic inflammation [36]. This makes the intestine one of the effective targets for DKD treatment. Based on this premise, several centuries ago, intestinal lavage was employed to treat renal failure [37], and colon dialysis therapy is still currently used. Accordingly, with a deeper understanding of gut microbiota, it has been demonstrated that the DKD-induced increase in uremic toxin can be reduced by reconstructing the homeostasis of gut microbiota [34, 38].

## LIMITATION

Our findings should be interpreted with consideration of the following limitations: First, the constrained sample size may reduce the statistical power of our conclusion. Consequently, the observed association between gut microbiota alterations (particularly A. muciniphila dynamics) and DKD progression requires further validation through large-scale, multi-center clinical trials. Second, the inherent heterogeneity of gut microbiota, shaped by confounding factors, such as dietary patterns, medication use (e.g., antibiotics or hypoglycemic agents), and environmental variables, poses challenges in establishing standardized intervention protocols. In real-world settings, the absence of "idealized" patient profiles complicates long-term compliance with microbiota-targeted therapies, which may impact intervention efficacy. Finally, current analytical methodologies for characterizing microbial community functions and metabolic interactions remain technically limited. Our interpretations rely on these preliminary findings, underscoring the need for advanced multi-omics approaches (e.g., metatranscriptomics or metabolomics) to comprehensively elucidate microbiota-host crosstalk in DKD pathogenesis.

## CONCLUSION

Our study demonstrates that *A. muciniphila* plays a pivotal role in modulating gut-kidney axis homeostasis in DKD. Notably, the relative abundance of *A. muciniphila* was significantly reduced in both fecal samples from DKD patients and intestinal tissues of DKD mice, suggesting its potential as a microbial biomarker for disease progression. Exogenous supplementation of *A. muciniphila* attenuated renal dysfunction, as evidenced by the marked reduction in urinary protein excretion, serum blood urea nitrogen (BUN), and creatinine levels. Furthermore, this intervention substantially decreased circulating levels of uremic toxins, including TMAO, p-cresol sulfonate, and indole sulfonate, which are implicated in oxidative stress and endothelial damage. Mechanistically, *A. muciniphila* restored gut microbial ecology by enriching short-chain fatty acid (SCFA)-producing taxa, thereby elevating luminal concentrations of acetate, propionate, and butyrate. These SCFAs may synergistically enhance intestinal barrier integrity by upregulating the expression of Muc2, occludin, and ZO-1, critical components of the mucus layer and tight junctions. This barrier reinforcement likely mitigated gut-derived endotoxin translocation and subsequent systemic inflammation, as reflected by reduced pro-inflammatory cytokines in treated mice. These findings position *A. muciniphila* as a promising therapeutic candidate for DKD, with dual benefits of uremic toxin clearance and renal protection via gut microbiota remodeling. Collectively, our work underscores the therapeutic potential of targeting gut microbiota dysbiosis to decelerate DKD progression, offering a novel paradigm for precision microbiome medicine in metabolic nephropathy.

## Figures and Tables

**Table 1:** The demographic characteristics of included patients.

-	Healthy Control	T2D	DKD
N	12	12	12
Age	50.36±5.48	51.62±6.13	50.84±6.06
Diabetes course	4.23±1.32	4.68±1.44	5.16±1.73
BMI (kg/m^2^)	21.64±2.06	21.23±2.15	22.03±2.22
Sex (M/F)	7/5	6/6	8/4
FPG (mmol/L)	6.4±0.46	7.0±0.43^**^	6.9±0.67
eGFR (mL/min/1/.73m2)	98.6±5.24	83.±4.09^**^	66.±4.79^**^^,##^

**Note: ** **p<0.01, *vs* healthy control; ^##^, **p<0.01, *vs* T2D.

**Abbreviation:** FPG, fasting plasma glucose; eGFR, estimated glomerular filtration rate.

**Table 2:** The glomerulus injury in DKD mice with or without supplemented with *A. muciniphila*.

Proportion of Glomerulus Lesions (%)					
Groups	N	No Lesions	Segmented Sclerosis	Diffuse Sclerosis	Exudative Lesion
C57BL/6J mice	8	93.6±3.53	6.4±2.48	0±0	0±0
Model-DKD	8	42.0±4.38^**^	38.0±4.66^**^	8.8±4.32	11.2±3.8
DKD-AKK	8	62.2±6.39^##^	22.4±5.88^##^	6.23±4.03	9.17±2.79

**Note: ** **, *p*<0.01, *vs* C57BL/6J mice; ^##^, *p*<0.01, *vs* DKD-AKK.

**Table 3:** The renal tubule injury in DKD mice with or without supplemented with *A. muciniphila*.

Renal Tubule Injury(%/ (N))							
Groups	N	Absent	Mild	Moderate	Severe	χ2	*p*
C57BL/6J mice	8	83 (5)	17 (1)	0	0		
Model-DKD	8	0 (0)	0 (0)	37.5 (3)	62.5 (5)	14.00	0.0029^*^
DKD-AKK	8	0 (0)	62.5 (5)	25 (2)	12.5 (1)	6.67	0.036^#^

Note: *, *p*<0.05, compared to C57BL/6J mice; #, *p*<0.05, *vs* DKD-AKK.

## Data Availability

Data are available from the NCBI with BioProject ID PRJNA1005777 and BioSample with accessions of SAMN36999167 to SAMN36999196.

## LIST OF ABBREVIATIONS

DKD: Diabetic Kidney Disease
SCFAs: Short-Chain Fatty Acids
ZO-1: Zona Occludens 1
MWAS: Metagenome-Wide Association Study
PBS: Phosphate-Buffered Saline
